# Plasma Membrane Proteolipid 3 Protein Modulates Amphotericin B Resistance through Sphingolipid Biosynthetic Pathway

**DOI:** 10.1038/srep09685

**Published:** 2015-05-12

**Authors:** Vinay K. Bari, Sushma Sharma, Md. Alfatah, Alok K. Mondal, K. Ganesan

**Affiliations:** 1 CSIR-Institute of Microbial Technology, Sector 39-A, Chandigarh – 160036, India

## Abstract

Invasive opportunistic fungal infections of humans are common among those suffering from impaired immunity, and are difficult to treat resulting in high mortality. Amphotericin B (AmB) is one of the few antifungals available to treat such infections. The AmB resistance mechanisms reported so far mainly involve decrease in ergosterol content or alterations in cell wall. In contrast, depletion of sphingolipids sensitizes cells to AmB. Recently, overexpression of *PMP3* gene, encoding plasma membrane proteolipid 3 protein, was shown to increase and its deletion to decrease, AmB resistance. Here we have explored the mechanistic basis of *PMP3* effect on AmB resistance. It was found that ergosterol content and cell wall integrity are not related to modulation of AmB resistance by *PMP3*. A few prominent phenotypes of *PMP3* delete strain, namely, defective actin polarity, impaired salt tolerance, and reduced rate of endocytosis are also not related to its AmB-sensitivity. However, *PMP3* overexpression mediated increase in AmB resistance requires a functional sphingolipid pathway. Moreover, AmB sensitivity of strains deleted in *PMP3* can be suppressed by the addition of phytosphingosine, a sphingolipid pathway intermediate, confirming the importance of this pathway in modulation of AmB resistance by *PMP3*.

Fungi cause superficial and invasive infections. Opportunistic invasive infections, though less prevalent, are of much greater concern because of high mortality (often over 50%) associated with them^1^. Many fungal species are responsible for these invasive infections, killing over one and half a million people every year, which is higher than that due to tuberculosis or malaria^1^. The treatment options for invasive infections are quite limited^2^. Amphotericin B (AmB) is a commonly used antifungal for over five decades. In spite of its toxicity, it is preferred for its broad-spectrum and fungicidal mode of action, particularly for treating invasive infections. Though echinocandins are also used for treating such infections, their use is limited in resource poor settings due to high cost. Moreover, *Cryptococcus* species do not respond to echinocandins and thus AmB alone (or in combination with flucytosine) is the mainstay to treat invasive infections caused by these species^2^,^3^.

AmB is currently considered to kill fungi by forming large, extramembranous fungicidal sterol sponge that depletes ergosterol from lipid bilayers^4^. Leakage of intracellular ions due to pore formation is thought to be a secondary effect of AmB^5^. Though AmB resistance is rare, it is seen in a significant percentage of pathogenic *Candida* species and filamentous fungi^6^,^7^. The AmB resistance mechanisms reported so far mainly involve reduction in ergosterol content or alterations in cell wall^7^,^8^,^9^,^10^,^11^. We have recently shown that sphingolipids also modulate AmB resistance^12^. A better understanding of AmB resistance/sensitivity mechanisms would facilitate developing therapeutic strategies to minimize evolution of AmB resistance, or to sensitize fungi to AmB such that lower AmB dose can be used to reduce toxicity.

While investigating apparent elevated AmB resistance of yeast cells in presence of farnesol (unpublished), we identified *Saccharomyces cerevisiae* *PMP3* gene as conferring increased AmB resistance when present in a multicopy plasmid. Deletion of this gene rendered the cells hypersensitive to AmB. During the course of our studies, *PMP3* gene's role in AmB resistance was also reported by Huang *et al*^13^, but the mechanism underlying this phenotype was not clear. *PMP3* was first reported as a non-essential gene whose deletion results in plasma membrane hyperpolarization and salt sensitivity^14^. It encodes a 55 amino acid hydrophophic protein of plasma membrane. A homologous plant protein could complement salt sensitivity of a yeast strain deleted in *PMP3*^15^. Here we have explored the mechanistic basis of *PMP3* effect on AmB resistance. We show that certain prominent phenotypes of *PMP3* delete strain, namely defects in salt tolerance, actin polarity and endocytosis, are not responsible for AmB-sensitivity of this strain. Instead, we demonstrate that modulation of AmB resistance by *PMP3* is mediated through sphingolipid biosynthetic pathway.

## Results and Discussion

### *PMP3* modulates AmB resistance

**Figure 1:**
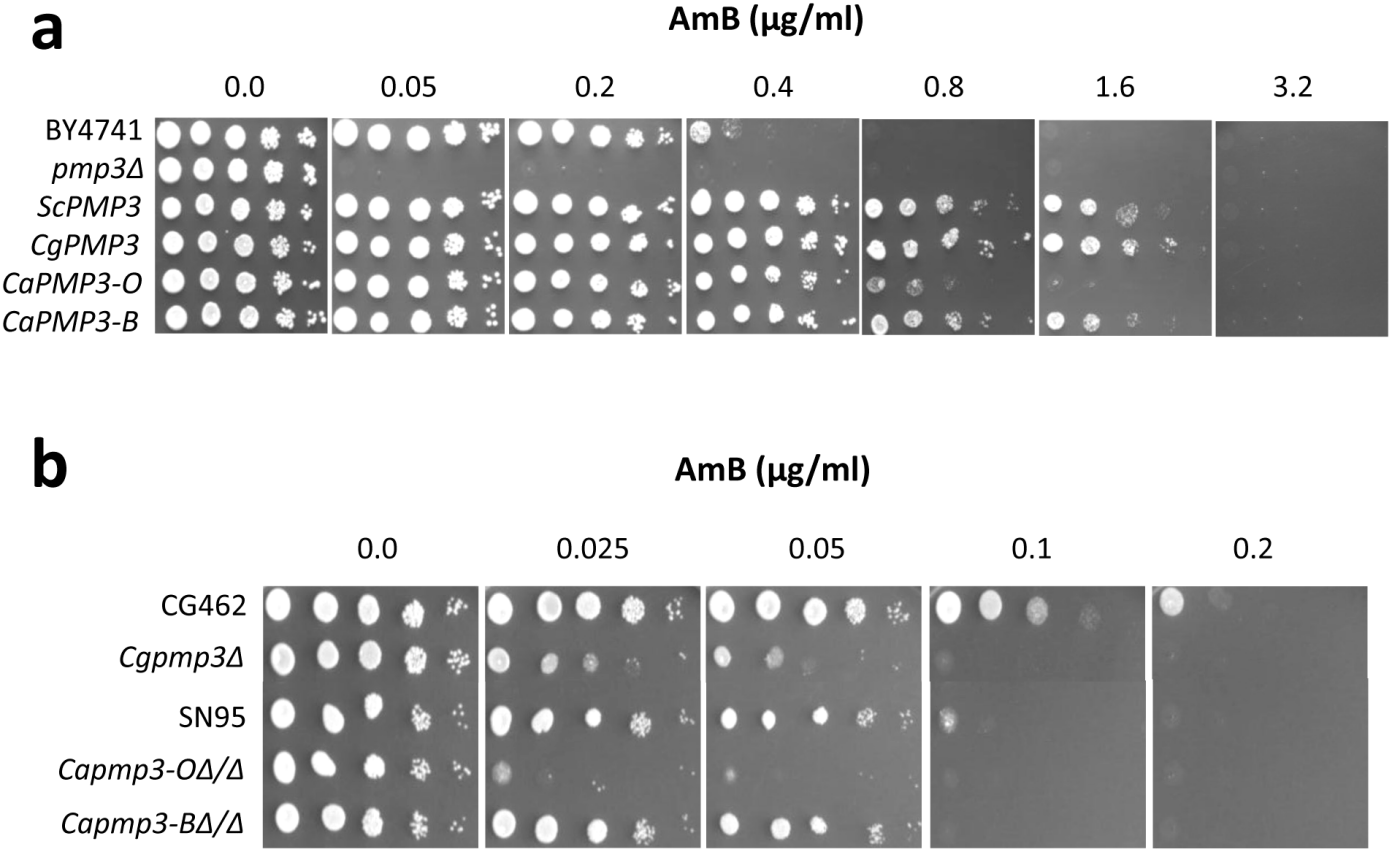
*S. cerevisiae* *PMP3* and its homologs from *C. albicans* and *C. glabrata* modulate AmB resistance. (a) Multicopy overexpression of *S. cerevisiae PMP3* (*ScPMP3*) and its homologs from *C. glabrata* (*CgPMP3*) and *C. albicans* (*CaPMP3-O*: *PMP3* ortholog, orf19.1655.3; *CaPMP3-B*: *PMP3* best hit, orf19.2959.1) in *pmp3Δ* strain of *S. cerevisiae* enhance AmB resistance by about 4-fold with respect to wild-type strain (BY4741) and about 32-fold with respect to *pmp3Δ* strain. The relative growth of the strains on 0.1 μg/ml AmB (not shown) was comparable to that of respective strains on 0.2 μg/ml AmB. (b) AmB sensitivity of *C. glabrata* strain deleted in *PMP3* ortholog (*Cgpmp3Δ*) and *C. albicans* strains deleted in both alleles of *PMP3* ortholog (*Capmp3-OΔ/Δ*) and *PMP3* best hit (*Capmp3-BΔ/Δ*), with respect to their respective parent strains CG462 and SN95. Five μl of 10-fold serial dilutions of cells were spotted starting from about 10^5^ cells/spot, as described in Methods.

The *S. cerevisiae* *PMP3* gene was isolated from a multicopy overexpression library (in plasmid pFL44L) as conferring higher resistance to AmB. A *PMP3* clone with 165 bp ORF along with 1196 bp upstream and 275 bp downstream regions was used in further studies. To confirm the phenotype, *PMP3* deletion and overexpression strains were compared with their parent strain for AmB resistance (Fig. 1a). While the delete strain was 8-fold more sensitive to AmB than the parent strain, the overexpression strain was about 4-fold more tolerant compared to the parent strain. During the course of this study, Huang *et al*^13^, while establishing a functional variomics tool for discovering drug-resistance genes and drug targets, also identified *PMP3* as conferring AmB resistance when present at more than one copies. *PMP3* (also known as *SNA1*) has three paralogs in *S. cerevisiae*, namely *SNA2*, *SNA3* and *SNA4*, which encode proteins with 40%, 34% and 41% identity, respectively, to that of *PMP3*^16^. Deletants of these genes were comparable to the parent strain in their susceptibility to AmB (results not shown), implying that these genes do not have any role in this phenotype.

To test if *PMP3* has a similar role in pathogenic yeasts, we searched for homologs in *C. albicans* and *C. glabrata*. *C. albicans* has two homologs, which encode proteins that show 51% and 45% identity at amino acid level to that of *S. cerevisiae*. The first one is referred to as *CaPMP3* ortholog (orf19.1655.3) and the second one as *CaPMP3* best hit (orf19.2959.1) in Candida Genome Database^17^. *C. glabrata* has a single ortholog *CgPMP3* (CAGL0M08552g) encoding a protein with 76% identity to ScPmp3p. The open reading frames of these homologs were PCR amplified and used to replace the ORF in *ScPMP3* clone, thereby placing these ORFs under the control of *ScPMP3* promoter and terminator in pFL44L vector. These were tested for their ability to modulate AmB resistance after being transformed into *pmp3*Δ strain of *S. cerevisiae*. *PMP3* ortholog from *C. albicans* was earlier shown to increase AmB resistance of *S. cerevisiae*^13^. In addition, we found *CaPMP3* best hit and *CgPMP3*, besides complementing *pmp3* mutation, provided resistance higher than that of wild-type strain (Fig. 1a). While the AmB resistance conferred by *CgPMP3* and *CaPMP3* best hit (*CaPMP3-B*) was similar to that of *ScPMP3*, i.e., 4-fold higher than that of wild-type strain, the *CaPMP3* ortholog (*CaPMP3-O*) provided 2-fold higher resistance (Fig. 1a).

To study the role of *CaPMP3* ortholog and *CaPMP3* best hit in *C. albicans*, we deleted both alleles of these genes in strain SN95 and confirmed by diagnostic PCR (Fig. S1). The *C. glabrata* ortholog *CgPMP3* (CAGL0M08552g) was also deleted and confirmed by diagnostic PCR (Fig. S2). The AmB susceptibility of these delete strains with respect to their parent strains was compared (Fig. 1b). While deletion of *PMP3* orthologs in *C. glabrata* and *C. albicans* sensitized the cells to AmB by about 4-fold, deletion of *CaPMP3* best hit did not have any effect. The AmB sensitivity of ortholog deletants in both these species provides strong evidence that *PMP3* gene is important for modulation of AmB resistance in pathogenic fungi as well.

### AmB resistance mediated by Pmp3p is not dependent on ergosterol or Hsp90 or cell wall integrity

As far as the mechanistic basis of *PMP3* effect on AmB resistance is concerned, Huang *et al*^13^ showed that it is not related to its role in ion homeostasis. Absence or severe reduction in the amount of ergosterol in the fungal membranes and its replacement with certain other sterols results in AmB resistance in fungi^7^,^10^,^11^. To address this possibility total cellular content of ergosterol was estimated, as described^18^. The ergosterol content, as % wet weight of cells, of parent, delete and overexpression strains, was 0.021 ± 0.001, 0.023 ± 0.002 and 0.023 ± 0.001, respectively. Though these values are comparable, it is possible that the intracellular distribution of ergosterol might be affected. To check this, cells were stained with filipin, which is specific for sterols^19^, and observed (Fig. S3a). While wild-type and *PMP3* overexpression strains showed intense fluorescent spots within cells, *pmp3Δ* strain lacked such spots. Thus, it is possible that more ergosterol is distributed in the plasma membrane of the delete strain, rendering it more accessible for AmB binding and killing. If this is true, then the delete strain should be more sensitive to other polyenes which also act by binding to ergosterol. However, the sensitivity *pmp3Δ* strain to the polyenes nystatin, natamycin and filipin was found to be comparable to that of wild-type and *PMP3* overexpression strains (Fig. S3b), ruling out ergosterol distribution or content having any role in modulation of AmB resistance by *PMP3*. Huang et al^13^ have also ruled out the involvement of ergosterol in modulation of AmB resistance by *PMP3*, since this gene did not affect the resistance against other polyenes.

A recent report suggested that AmB resistance of ergosterol biosynthetic pathway mutants is highly dependent on Hsp90 chaperone and these mutants are hypersensitive to Hsp90 inhibitors radicicol and geldanamycin as well as oxidative stress^20^. To check the Hsp90 dependence of AmB resistance conferred by *PMP3*, the sensitivity of this strain to radicicol and oxidative stress was checked along with *erg6Δ* strain as positive control (Table 1). The AmB resistance of *erg6Δ* strain and *PMP3* overexpression strain was comparable. However, while *erg6Δ* strain was 8-fold and 4-fold, respectively, more sensitive to radicicol and oxidative stress, the sensitivity of *PMP3* overexpression strain was comparable to wild-type, implying that Pmp3p is not dependant on Hsp90 for conferring AmB resistance. Cell wall alterations also can affect AmB resistance^7^. Compared to parent strain, *PMP3* delete strain showed normal chitin deposition (Fig. S4a), as well as similar resistance to cell wall disrupting agents calcofluor white, sodium dodecyl sulphate and congo red (Fig. S4b), implying that AmB sensitivity of delete strain is not related to cell wall integrity.

### Actin polarity and endocytosis, though impaired in *pmp3Δ* strain, are not responsible for its AmB sensitivity

To gain further insight into *PMP3* mechanism of action, we tried to predict its possible functions on the basis of biological roles of genes that interact with *PMP3*. The list of interacting genes was analyzed using DAVID Bioinformatics Resources^21^ for enrichment of gene ontology terms for biological processes. The top-two annotation clusters corresponded to endocytosis and actin cytoskeleton (Table 2). To check if impaired endocytosis would result in AmB sensitivity, we screened mutants of several genes having role in endocytosis for their AmB sensitivity. Deletants of *RVS161* and *RVS167* were about 4-fold more sensitive to AmB compared to the parent strain (Fig. S5). These strains, besides defects in endocytosis have several other phenotypes including salt sensitivity and altered actin cytoskeleton^22^,^23^,^24^,^25^. *SUR7*, encoding an eisosome protein involved in endocytosis, partially suppresses several of these phenotypes upon multicopy overexpression^26^,^27^,^28^. Thus, we exploited overexpression of *SUR7* to understand if AmB sensitivity of *pmp3*Δ strain is a consequence of defects in actin cytoskeleton or endocytosis, or it is an independent phenotype.

**Figure 2:**
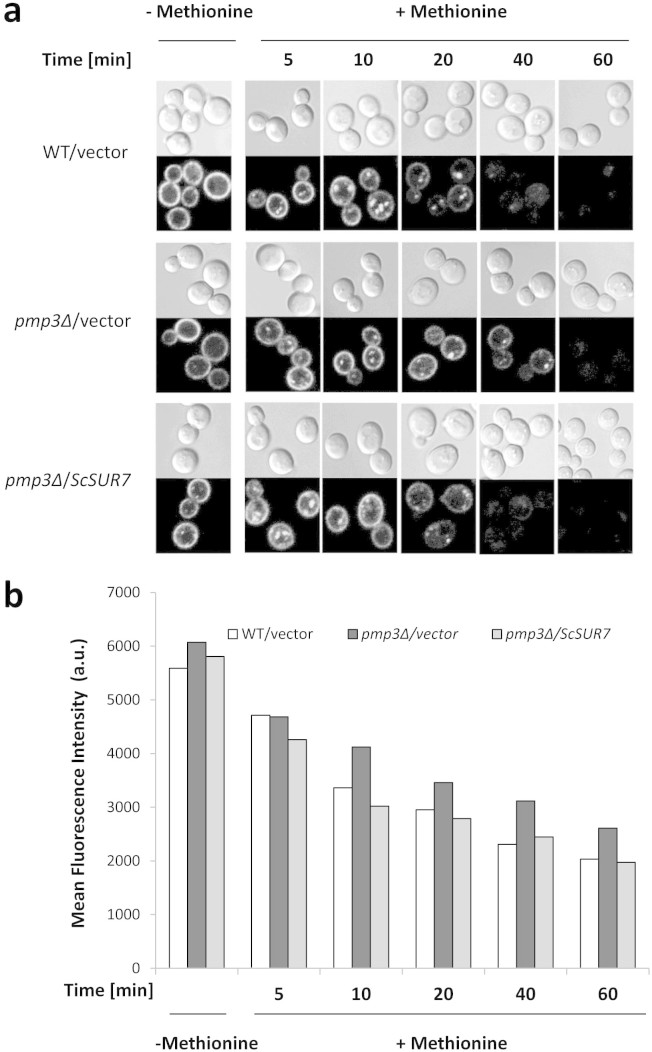
Slow rate of endocytosis of *pmp3Δ* strain is restored to normal level by overexpression of *ScSUR7*. (a) Wild type strain 3818 (SEY6210-Mup1pHluorin) and *pmp3Δ* strain (3818 *pmp3Δ*::*HIS3*) transformed with either vector or *ScSUR7*, were grown without methionine to promote accumulation of Mup1-pHluorin in the plasma membrane. After addition of 20 μg/ml methionine, random fields of cells were imaged at different time intervals. All images were obtained at identical exposure conditions. (b) After addition of methionine, Mup1-pHluorin fluorescence was measured at indicated time intervals in a flow cytometer, as described in Methods. The values shown are average of two replicates from one representative experiment. Experiments were repeated thrice with comparable results.

A large scale survey using GFP-Snc1-Suc2 reporter has indicated that endocytosis is decreased in *pmp3*Δ strain^29^. We monitored rate of endocytosis with a different reporter, namely methionine permease (Mup1) tagged with ecliptic pHluorin, which is a pH-sensitive green fluorescent protein variant that does not fluoresce after internalization to an acidic compartment like vacuole^30^,^31^. Mup1-pHluorin is internalized rapidly upon exposure to methionine. Wild-type cells showed substantial decrease in Mup1-pHluorin intensity within 20 min after adding methionine (Fig. 2a). However, in *pmp3Δ* strain 40 min was needed for a similar decrease, confirming that the rate of endocytosis is slowed down in this strain. *SUR7* expressed from a multicopy plasmid restored the rate of endocytosis of *pmp3Δ* strain to normal level (Fig. 2a). Mup1-pHluorin fluorescence was also monitored by flow cytometry (Fig. 2b). Though background fluorescence was high for all the strains, the rate of decrease in fluorescence is indicative of rate of endocytosis. While it was slow in the *pmp3Δ* strain, it was restored to wild-type level upon *SUR7* overexpression.

**Figure 3:**
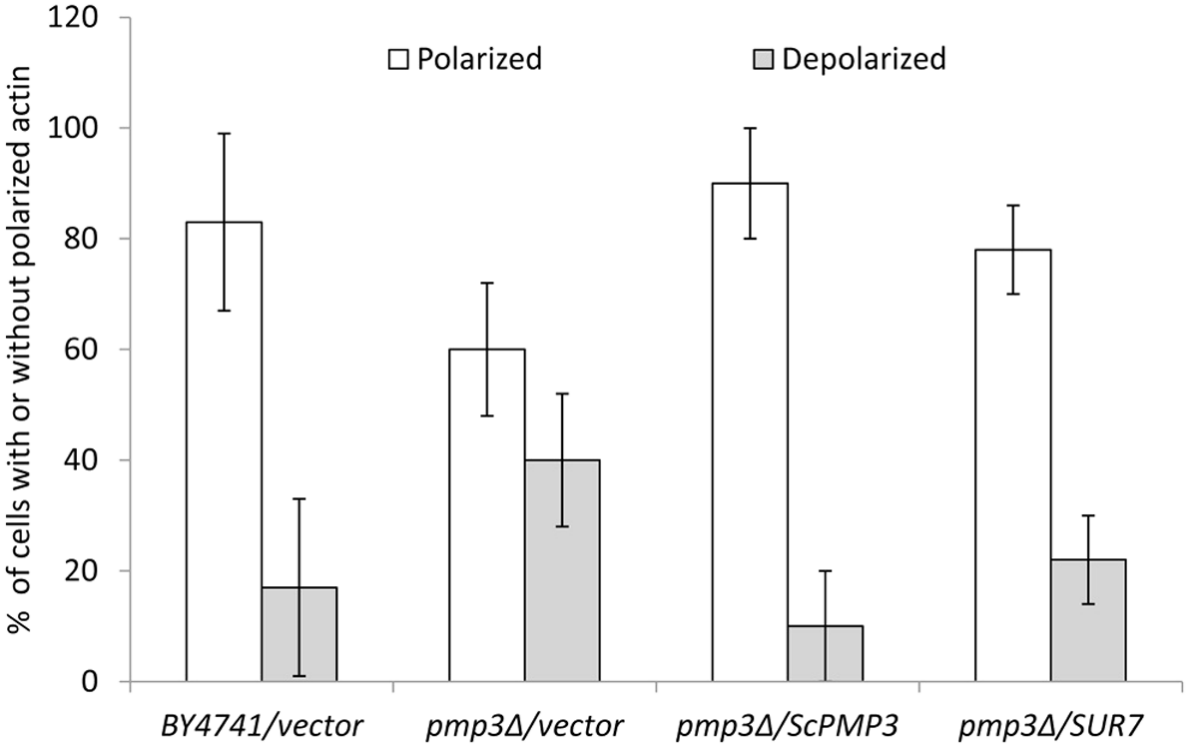
Actin polarization defect of *pmp3Δ* strain is suppressed by multicopy *SUR7* overexpression. Cells were grown to log phase and actin was visualized by rhodamine phalloidin staining. About 200 cells with small buds were scored according to their polarization state. Cells with actin patches concentrated in the small bud, with fewer than four patches in the mother cell, were classified as polarized cells. Other cells with more actin patches in the mother cell than in the small bud were classified as depolarized cells. Representative images are shown in Figure S6. Mean values of two independent experiments are given. The error bars indicate the range.

**Figure 4:**
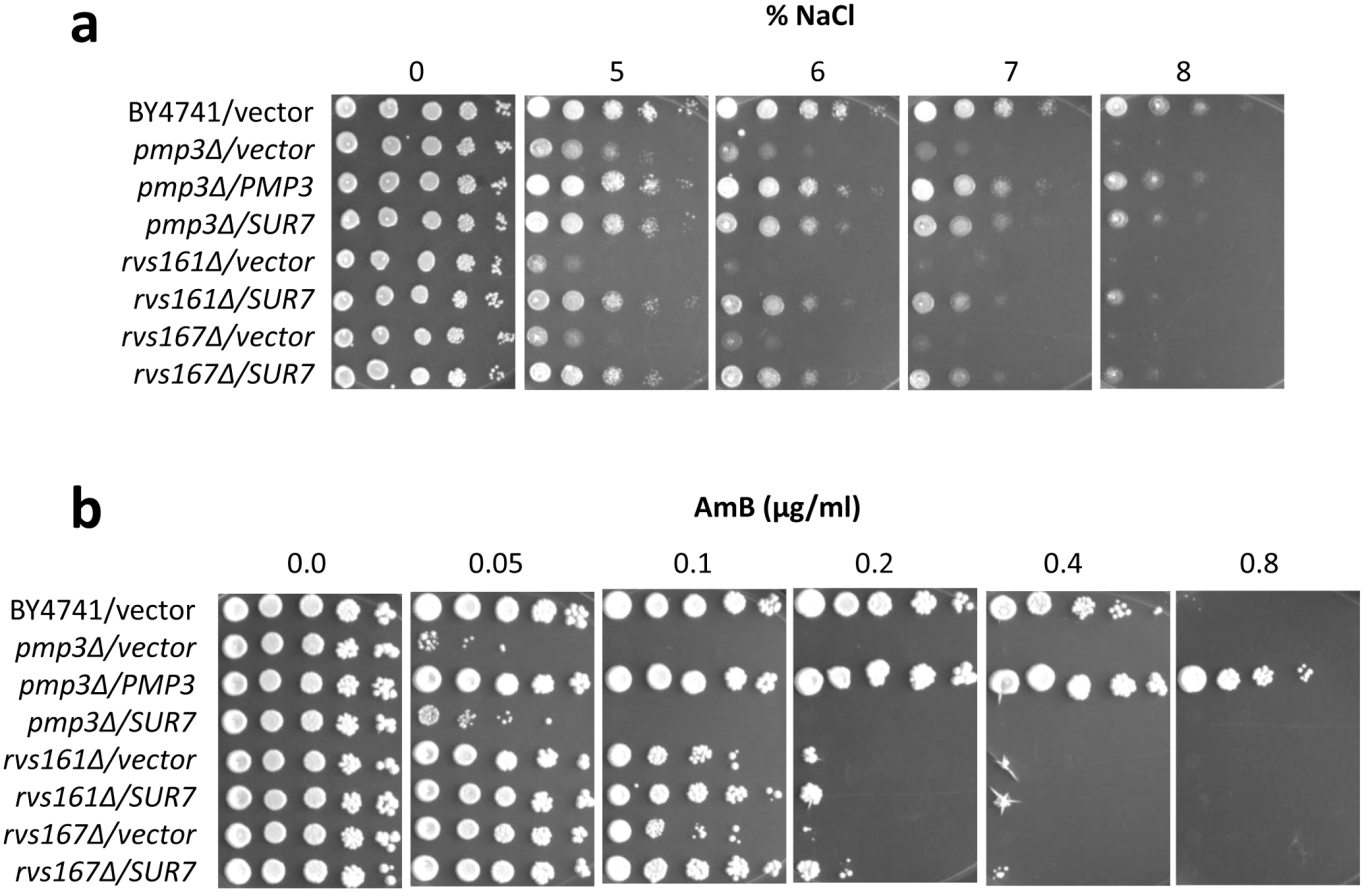
*SUR7* overexpression can suppress salt sensitivity (a), but not AmB sensitivity (b) of strains deleted in *PMP3, RVS161* or *RVS167*. Wild-type (BY4741) and *PMP3* overexpression strains are included as controls.

Actin cytoskeleton plays a central role in endocytosis^25^ and *rvs161*Δ and *rvs167*Δ strains impaired in endocytosis also have actin polarization defects^23^. Moreover, as *PMP3* interacts with genes having role in actin cytoskeleton (Table 2), we visualized actin in *PMP3* strains. The *pmp3*Δ strain showed pronounced defect in actin polarity, which is suppressed by overexpression of *SUR7* (Fig. 3 and Fig. S6). *SUR7* also suppressed the sensitivity of *pmp3*Δ, *rvs161*Δ and *rvs167*Δ strains to NaCl (Fig. 4a). However, it could not reverse the sensitivity of these strains to AmB (Fig. 4b), demonstrating that AmB sensitivity of these mutants is not mediated by defects in actin polarity, endocytosis or NaCl tolerance.

### Sphingolipid biosynthetic pathway is essential for *PMP3* mediated increase in AmB resistance

**Figure 5:**
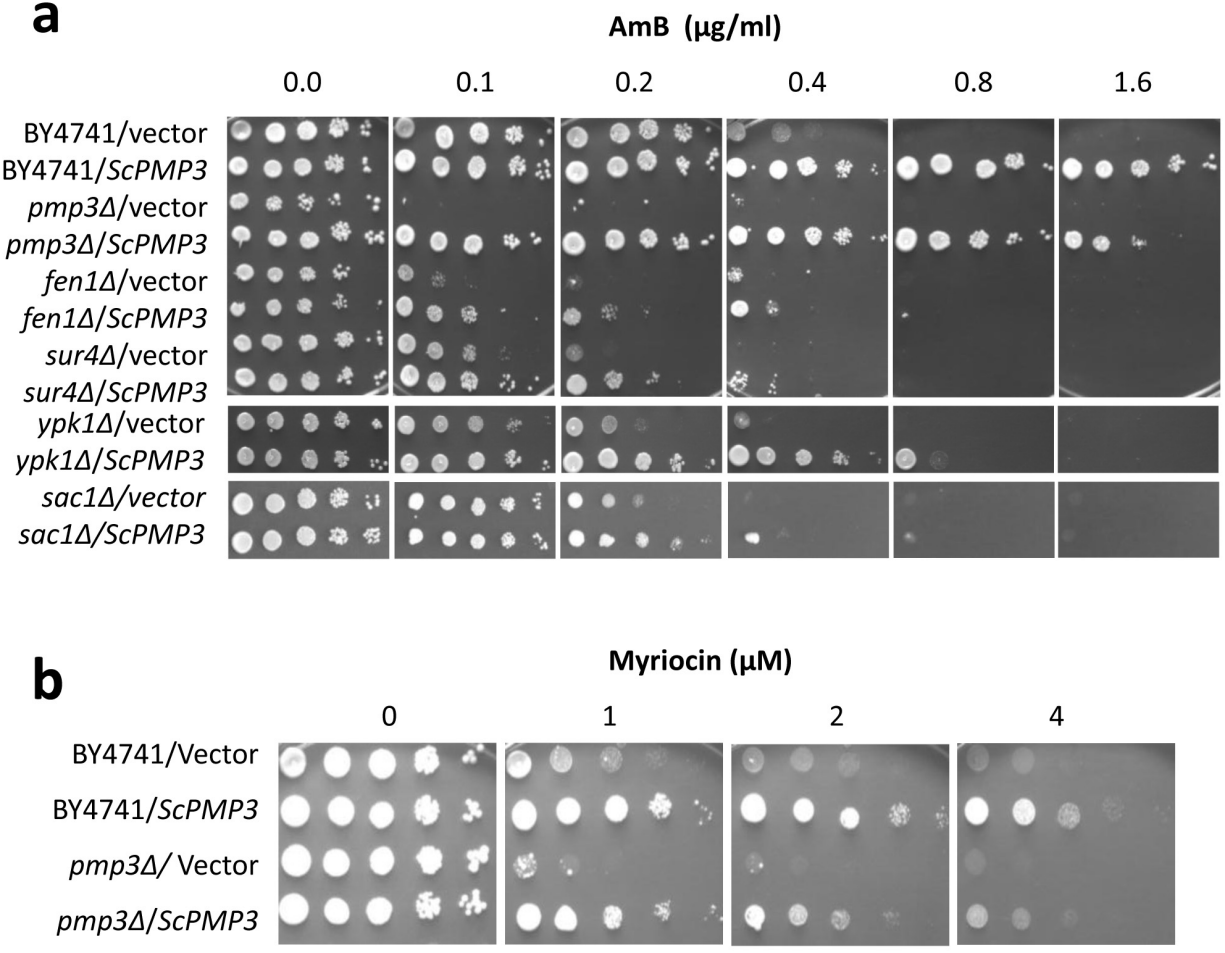
*PMP3* modulates AmB resistance through sphingolipid biosynthetic pathway. (a) Sphingolipid biosynthetic pathway genes *FEN1* *and SUR4* and regulatory genes *YPK1* and *SAC1* are important for *PMP3* mediated increase in AmB resistance. Wild-type (BY4741) and *pmp3Δ* strains overexpressing *ScPMP3* serve as positive controls. (b) *PMP3* modulates tolerance to myriocin, a sphingolipid biosynthetic pathway inhibitor. While strains overexpressing *PMP3* are about 4-fold more tolerant, the strain deleted in *PMP3* is about 2-fold more sensitive to myriocin, compared to the wild-type strain BY4741.

We had recently shown that sphingolipid biosynthetic pathway genes *FEN1* (*ELO2*) and *SUR4* (*ELO3*) modulate AmB resistance^12^. While inhibition of sphingolipid biosynthesis with myriocin sensitized cells to AmB, addition of phytosphingosine, a sphingolipid pathway intermediate, reversed this phenotype^12^. To check the importance of this pathway for *PMP3* mediated increase in AmB resistance, multicopy *ScPMP3* was transformed into a few sphingolipid pathway mutants and the resistance was checked (Fig. 5a). In the wild-type parent strain (BY4741) *ScPMP3* could increase AmB resistance at least by 4-fold. However, it increased AmB resistance by 2-fold or less in mutants of sphingolipid biosynthetic genes *FEN1* and *SUR4*, and regulatory genes *YPK1*^32^,^33^ and *SAC1*^34^. If *PMP3* overexpression effect is independent of sphingolipid pathway, then fold-increase in AmB resistance by *PMP3* in these mutants should have been comparable to that of the parent strain. Only 2-fold or less increase in resistance shows that *PMP3* is dependent on this pathway for enhancing AmB resistance. Even this increase appears to be due to genetic redundancy. *FEN1* and *SUR4* are involved in fatty acid elongation and can partly compensate for each other's loss, since double deletion is lethal^35^. *YPK1* and *YPK2* are synthetic lethal^36^ and arose from the whole genome duplication^37^. Sac1p is a phosphatidylinositol phosphate phosphatase, and its catalytic domain (Sac1-like domain) is seen among several phosphatases with partially overlapping function^38^. Sac1p is known to modulate sphingolipid metabolism^34^,^39^. Physical interaction of Pmp3p and Sac1p has also been reported in a large-scale study^40^. Thus it appears likely that Pmp3p modulates sphingolipid biosynthesis and AmB resistance by interacting with Sac1p. Dependence of Pmp3p on Sac1p provides possible link between Pmp3p and sphingolipid pathway.

**Figure 6:**
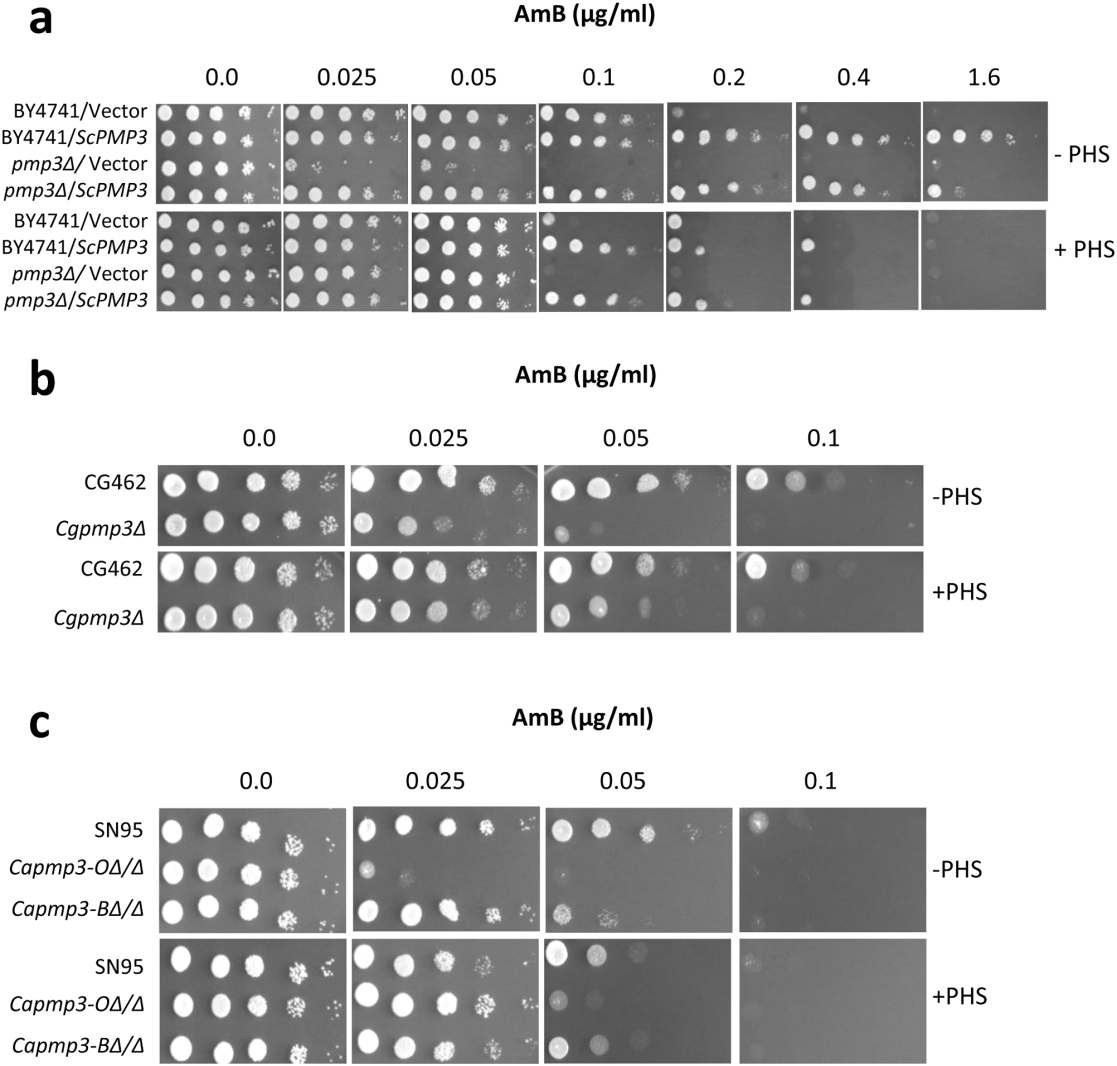
Phytosphingosine (PHS), a sphingolipid pathway intermediate, modulates AmB resistance. (a) Growth of wild-type (BY4741), *PMP3* deletion and overexpression strains of *S. cerevisiae* on indicated concentrations of AmB alone or in combination with 5 μM phytosphingosine (PHS). Relative growth of strains at 0.8 μg/ml AmB (not shown) was comparable to their growth at 1.6 μg/ml. (b) Growth of wild-type (CG462) and *PMP3* delete (*Cgpmp3Δ*) strains of *C. glabrata* on indicated concentrations of AmB alone or in combination with 5 μM PHS. (c) Growth of *C. albicans* strains deleted in both alleles of *PMP3* ortholog (*Capmp3-OΔ/Δ*) or *PMP3* best hit (*Capmp3-BΔ/Δ*), with respect to their parent SN95 on indicated concentrations of AmB alone or in combination with 10 μM PHS.

Myriocin inhibits the first committed step of sphingolipid biosynthesis catalyzed by serine palmitoyltransferase^33^. Sphingolipid pathway regulatory genes *YPK1*^32^,^33^ and *SAC1*^34^ modulate myriocin resistance. To test if *PMP3* also regulates sphingolipid pathway, we checked myriocin resistance of deletion and overexpression strains. While deletion of *PMP3* decreased myriocin resistance by 2-fold, its overexpression increased myriocin resistance by 4-fold, both with respect to parent strain (Fig. 5b), indicating that *PMP3* is possibly involved in regulation of this pathway in *S. cerevisiae*. We also checked the myriocin sensitivity of *C. glabrata* strain deleted in *PMP3* ortholog, and *C. albicans* strains deleted in *PMP3* ortholog or best hit. However, the sensitivity of these strains was found to be comparable to that of their respective parent strains (Fig. S7). Another approach used to establish the role or dependence of genes on sphingolipid pathway is by supplementing with phytosphingosine (PHS), a sphingolipid pathway intermediate^33^,^41^. Addition of PHS increased the AmB resistance of *pmp3Δ* strain of *S. cerevisiae* to wild type level. It also decreased the AmB resistance of *PMP3* overexpression strain to nearly wild type level (Fig. 6a), perhaps by its known antifungal activity at high concentration^42^. PHS also suppressed AmB sensitivity of *C. glabrata* and *C. albicans* strains deleted in *PMP3* orthologs (Figs. 6b and 6c). These results further establish that *PMP3* modulates AmB resistance through sphingolipid pathway in *S. cerevisiae* as well as in pathogenic *Candida* species.

Sphingolipid bases and complex sphingolipids have multiple roles in cells, both as structural components and as signalling molecules^43^,^44^. Mutants of sphingolipid pathway show pleiotropic phenotypes^44^, of which those affected in actin cytoskeleton^45^, endocytosis^46^ and AmB resistance^12^ are pertinent here. Since actin is critical for endocytosis^25^, defective endocytosis could be a consequence of impaired actin polarity. Thus, impaired actin cytoskeleton and slow rate of endocytosis of *pmp3*Δ strain are consistent with the regulatory role played by *PMP3* in sphingolipid pathway.

In conclusion, we have shown that a few striking phenotypes of *PMP3* mutant, such as impaired actin polarity, endocytosis and salt tolerance are not related to its AmB-sensitivity. Rather, we show that modulation of AmB resistance by *PMP3* is dependent on sphingolipid biosynthetic pathway, since AmB sensitivity of *PMP3* deletants is suppressed by phytosphingosine, a sphingolipid pathway intermediate. Moreover, enhanced AmB resistance conferred by overexpression of *PMP3* is dependent on functional sphingolipid biosynthetic and regulatory genes. Efforts are underway to elucidate the precise mechanism underlying *PMP3* effect or dependence on sphingolipid pathway for modulating AmB resistance.

## Methods

Fine chemicals and yeast synthetic drop-out medium supplements without uracil were procured from Sigma. All other media components were obtained from BD (Difco). Oligonucleotides were custom synthesised from Sigma-Genosys, India. Restriction enzymes, DNA polymerases and other DNA modifying enzymes were obtained from New England Biolabs, and DNA purification kits were obtained from Qiagen.

### Strains, media and growth conditions

*S. cerevisiae* and *Candida* strains and plasmids used in this study are listed in Table S1 and S2. The *Escherichia coli* strain DH5α was used as a cloning host. YPD and Synthetic complete (SC) media were prepared and used as described^12^. Uracil supplement is omitted in SC medium to provide SC-ura medium. Yeast transformations were carried out using the modified lithium acetate method^47^. Stock solutions of AmB (2 mg/ml), myriocin (5 mM), phytosphingosine (15 mM) and radicicol (5 mM) were prepared in DMSO. Stock solutions of nourseothricin (200 mg/ml) and tert-butyl hydroperoxide (500 mM) were made in water.

### Growth assays by dilution spotting

For dilution spotting assays, the strains/transformants were grown overnight in SC or SC-ura medium, reinoculated in fresh medium to an *A*_600_ of 0.1 and grown for 6 h. The exponential phase cells were harvested, washed and resuspended in sterile water to an *A*_600_ of 1.0 (~2 × 10^7^ cells/ml). Ten-fold serial dilutions were made in water and 5 μl of each dilution was spotted on SC or SC-ura plates with desired concentration of compounds, as mentioned in Figures. DMSO alone was included in control plates, corresponding to its concentration in experimental plates, where appropriate. Plates were incubated for 2 days at 30°C before taking photographs. These experiments were repeated at least three times with comparable results.

### Cloning methods

The ORFs of putative homologs of *ScPMP3* in *C. albicans* [*CaPMP3*-ortholog (orf19.1655.3), *CaPMP3*-Best hit (orf19.2959.1)] and *C. glabrata* (*CAGL0M08552g*) were PCR amplified from the genomic DNA of *C. albicans* and *C. glabrata* with specific primers sets (Table S3). The PCR products were then used to replace the *ScPMP3* ORF in a Sc*PMP3* clone in multicopy vector pFL44L, using Circular Polymerase Extension Cloning (CPEC) method^48^,^49^, thereby retaining the *ScPMP3* promoter and terminator regions for all *PMP3* orthologs as well. For cloning *ScSUR7* gene, the *SUR7* ORF of *S. cerevisiae* along with its promoter and terminator (+568 to −326 bp) was amplified from strain BY4741 with forward primer ScSUR7-OCS1 and reverse primer ScSUR7-OCA1 (Table S3) and cloned in pFL44L by CPEC method^48^,^49^.

### Construction of *C. albicans* strains deleted in *CaPMP3*-ortholog and *CaPMP3*-best hit

Both alleles of *CaPMP3*-ortholog (orf19.1655.3) and *CaPMP3*-Best hit (orf19.2959.1) were deleted in *C. albicans*, using *HAH2* cassette and gene-specific primers, as described^12^, and confirmed by diagnostic PCR with appropriate primers (Table S3).

### Construction of *C. glabrata* strain deleted in *CgPMP3*

*PMP3* ortholog in *C. glabrata* (*CAGL0M08552g*) was deleted using a selection cassette conferring nourseothricin resistance containing *CaNAT1* gene with codon usage adapted for *Candida* species^50^. A 508 bp region upstream of, and 472 bp region downstream of *CgPMP3* ORF were PCR amplified from wild type genomic DNA using primers for upstream (CgPMP3-US1 and CgPMP3-UA1) and downstream regions (CgPMP3-DS1 and CgPMP3-DA1). The upstream flanking region was fused with the 5′ region of *CaNAT1* cassette using amplified upstream region and plasmid (pCR2.1-NAT^51^) with *CaNAT1* as templates and primers CgPMP3-US1 and CaNAT1-US-R1 to generate upstream split marker. Similarly, the downstream flanking region was fused to 3′ region of *CaNAT1* cassette with amplified downstream region and pCR2.1-NAT as templates and primers CaNAT1-DS-F1 and CgPMP3-DA1 to generate downstream split marker. These fusion products, which share 401 bp homology between them in the cassette, were mixed together, transformed^52^ into *C. glabrata* wild type strain CG462, and plated on YPD plate. After incubation at 30°C for 24 h, cells were replica-plated onto YPD plate with 200 μg/ml nourseothricin and further incubated for 24 h. Nourseothricin resistant colonies were purified and checked for gene deletion by diagnostic PCR using cassette specific primers and primers outside the flanking region of homology (Table S3).

### Fluorescence microscopy

Mup1-pHluorin internalization assay was performed as reported^31^,^53^. Mup1- pHluorin localization was visualized using a Nikon A1R confocal microscope using FITC optics and 100X oil immersion objective. Images were analysed using NIS Elements software. Visualization of actin by rhodamine phalloidin staining was carried out as described^54^. Calcofluor staining of cell wall was done as described^55^. The subcellular localization of sterols was monitored by staining with filipin as described^19^ with slight modification. Exponentially growing cells (0.5 OD cells/ml) were fixed with 3.7% paraformaldehyde for 10 min at 30°C, washed with phosphate-buffered saline (PBS) and incubated with 5 μg/ml of filipin (Sigma F9765) in the dark at 30°C for 5 min. The stained cells were directly observed under a confocal laser scanning microscope (Nikon A1R) using 405 nm laser and images were analysed using NIS element software.

### Flow cytometry

Log-phase cells were grown in SC medium without uracil and methionine for 6 hours, and then methionine was added to 20 μg/ml final concentration. At different time intervals cells were collected by centrifugation, washed and resuspended in PBS. Mup1-pHluorin fluorescence was measured with BD Accuri™ C6 flow cytometer in FL1 channel. Excitation and emission wavelengths were 488 nm and 530 nm, respectively. For each sample 10^4^ cells were analysed. Three independent experiments were done with two replicates each time.

## Author Contributions

K.G. designed the project and provided overall guidance. V.K.B. and S.S. carried out the experiments and collected data. V.K.B. and K.G. drafted and finalized the manuscript. A.K.M. provided technical inputs and guidance for confocal microscopy. S.S., M.A. and A.K.M. provided critical input during group meetings and on the manuscript. All authors reviewed the manuscript.

## Supplementary Material

Supplementary Information Supplementary Information

## Figures and Tables

**Table 1:** AmB resistance mediated by *PMP3* is not dependant on *HSP90*

Strain	MIC		
	AmB, μg/ml	Radicicol, μM	TBH, mM
BY4741/vector	0.4	16	2
*pmp3Δ*/vector	0.05	8	2
*pmp3Δ*/*ScPMP3*	1.6	16	2
*erg6Δ*/vector	1.6	2	0.5

MIC for AmB and radicicol was determined in SC-ura broth at 30°C. Sensitivity to oxidative stress was determined by dilution spotting on SC-ura agar medium with tert-butyl hydroperoxide (TBH) at 37°C. MIC is the concentration at which no growth was observed.

**Table 2:** Functional Annotation Clustering of *PMP3* interacting genes

Cluster 1 - Enrichment Score: 2.82		
Term	Count	P-Value
GO:0006897 ~ endocytosis	12	2.07E-04
GO:0010324 ~ membrane invagination	13	1.32E-03
GO:0016192 ~ vesicle-mediated transport	23	4.18E-03
GO:0016044 ~ membrane organization	18	4.57E-03
*ACT1, AGE1, CMD1, CSR2, CYC2, ERG3, FEN1, GTS1, INP52, MGM1, MVB12, MYO5, PHO86, RIM8, RUD3, SHR3, SLA1, SSO2, SUR7, VAM10, VPS24, VPS27, VPS30, VPS8, VRP1, WHI2.*		

Cluster 2 - Enrichment Score: 2.40		
Term	Count	P-Value
GO:0006970 ~ response to osmotic stress	14	2.38E-05
GO:0048308 ~ organelle inheritance	10	2.89E-04
GO:0030036 ~ actin cytoskeleton organization	11	1.88E-03
GO:0008064 ~ regulation of actin polymerization or depolymerization	4	2.39E-03
GO:0032271 ~ regulation of protein polymerization	4	2.39E-03
GO:0030833 ~ regulation of actin filament polymerization	4	2.39E-03
GO:0030832 ~ regulation of actin filament length	4	2.39E-03
GO:0033043 ~ regulation of organelle organization	10	2.63E-03
GO:0043254 ~ regulation of protein complex assembly	5	2.76E-03
GO:0030029 ~ actin filament-based process	11	2.85E-03
GO:0032970 ~ regulation of actin filament-based process	4	5.84E-03
GO:0032956 ~ regulation of actin cytoskeleton organization	4	5.84E-03
GO:0031333 ~ negative regulation of protein complex assembly	3	9.69E-03
GO:0007010 ~ cytoskeleton organization	15	1.13E-02
GO:0044087 ~ regulation of cellular component biogenesis	5	1.16E-02
GO:0051493 ~ regulation of cytoskeleton organization	5	3.28E-02
GO:0032535 ~ regulation of cellular component size	9	3.42E-02
GO:0007015 ~ actin filament organization	5	1.50E-01
*ABP1, ACT1, ARC15, ARC18, BIM1, CAP1, CAP2, CDC13, CMD1, DFG16, EMP70, ENA1, GLC7, GTS1, HSC82, HSP82, INP52, IST2, KTI12, MGM1, MVB12, MYO5, NIP100, NST1, PET122, SHE4, SKY1, SLA1, VMA9, VRP1, WHI2.*		

List of *PMP3* interacting genes were downloaded from SGD^16^ and analyzed with DAVID Functional Annotation Clustering tool (http://david.abcc.ncifcrf.gov/home.jsp) of DAVID Bioinformatics Resources v6.7^21^ for enrichment of gene ontology terms using default parameters, but restricted to biological processes (GOTERM_BP_FAT category). Only the top-two annotation clusters with greater than 2-fold enrichment are listed, along with the genes grouped under each cluster.
