# Supplementary material for: Plasma Membrane Proteolipid 3 Protein Modulates Amphotericin B Resistance through Sphingolipid Biosynthetic Pathway
Source: Sci Rep. 2015 May 12;5:9685. doi: 10.1038/srep09685 (PMC4428271; doi:10.1038/srep09685)
Supplement: Supplementary Information [file srep09685-s1.pdf]

Supplementary Information:

Plasma Membrane Proteolipid 3 Protein Modulates Amphotericin B Resistance through Sphingolipid Biosynthetic Pathway

Vinay K. Bari, Sushma Sharma, Md. Alfatah, Alok K. Mondal and K. Ganesan

Contents:

Supplementary Figures S1 to S7

Table S1. List of *S. cerevisiae* and *Candida* strains

Table S2. List of plasmids

Table S3. List of oligonucleotides

References

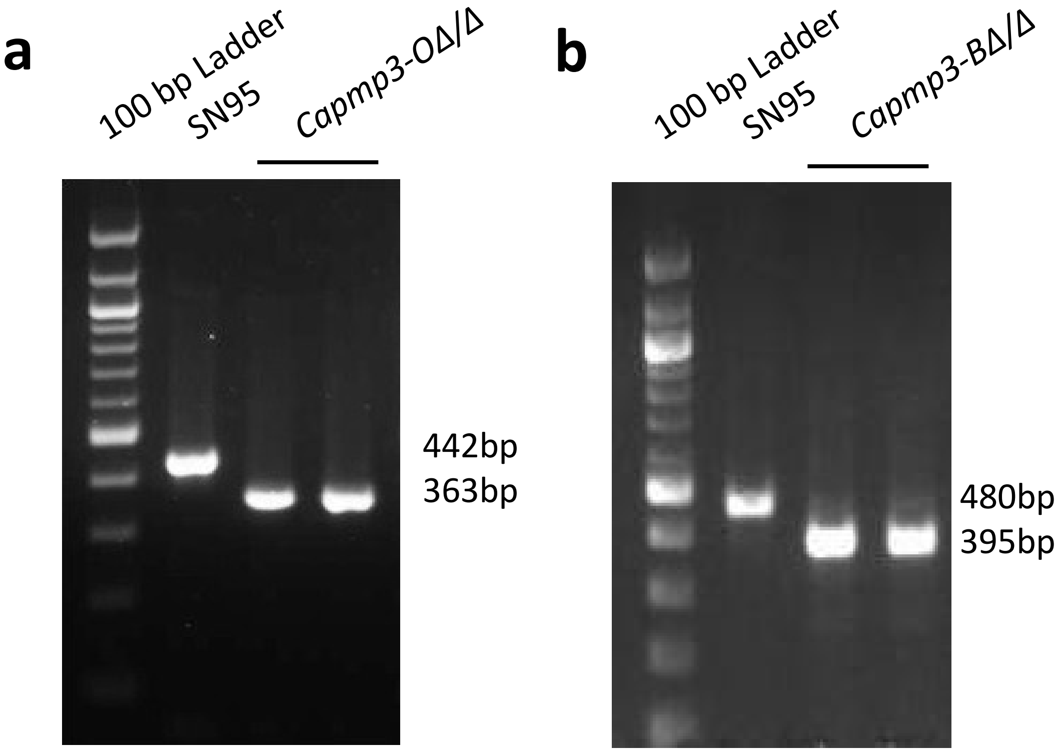

Figure S1. Diagnostic PCR to confirm deletion of *PMP3* ortholog and *PMP3* best hit in *C. albicans.* (**a**) Diagnostic PCR for deletion of *CaPMP3* ortholog was done with upstream forward (CaPMP3-O-S3) and downstream reverse (CaPMP3-O-A3) primers, external to flanking regions of homology used for targeted gene deletion. The size of the PCR products obtained correspond to the expected size, 442bp and 363bp, for parent strain SN95 and *Capmp3-OΔ/Δ,* respectively. (**b**) For checking deletion of *CaPMP3* best hit, primers (CaPMP3-B-S3 and CaPMP3-B-A3) external to flanking regions of homology used for targeted gene deletion were used. The size of the PCR products obtained correspond to the expected size, 480bp and 395bp, for SN95 and *CaPMP3-BΔ/Δ*, respectively.

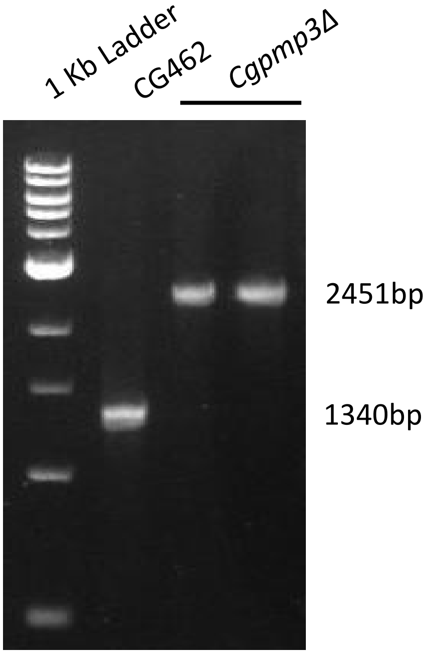

Figure S2. Diagnostic PCR for deletion of *PMP3* gene in *C. glabrata* was done with upstream forward (CgPMP3-DG-US2) and downstream reverse (CgPMP3-DG-DA2) primers, external to flanking regions of homology used for targeted gene deletion. The size of the PCR products obtained correspond to the expected size, 1340bp and 2451bp respectively, for parent strain CG462 and *Cgpmp3Δ* strain.

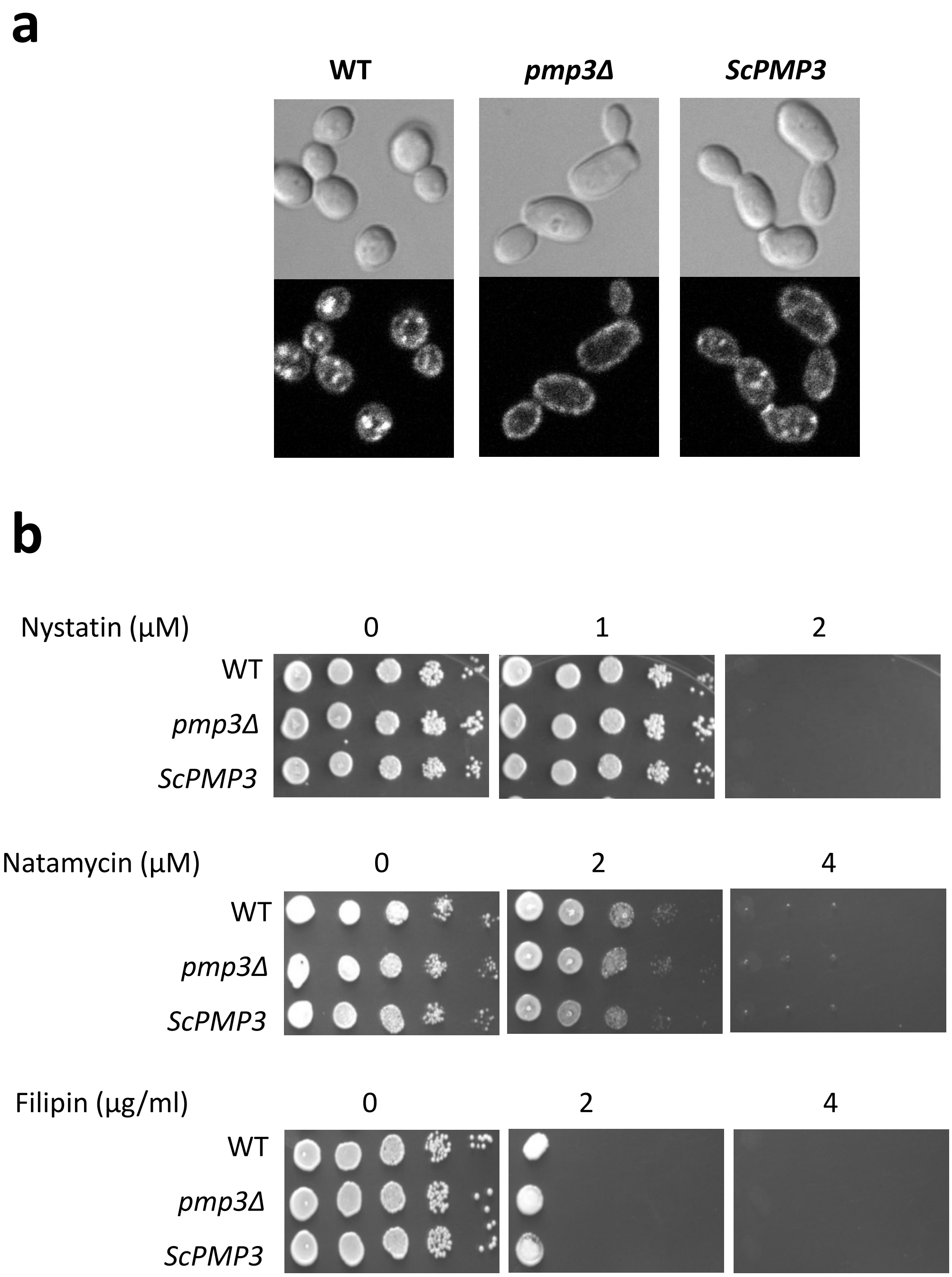

Figure S3. (**a**) Sterol distribution determined by filipin staining shows fluorescence associated with internal membranes in wild type (WT) and *ScPMP3* overexpression strains but not in *pmp3Δ* strain (bottom panels). Top panels show DIC images of cells. (**b**) *PMP3* gene does not modulate resistance to polyenes nystatin, natamycin and filipin. Growth of WT, *PMP3* delete and overexpression strains was assessed on plates containing indicated concentration of polyenes.

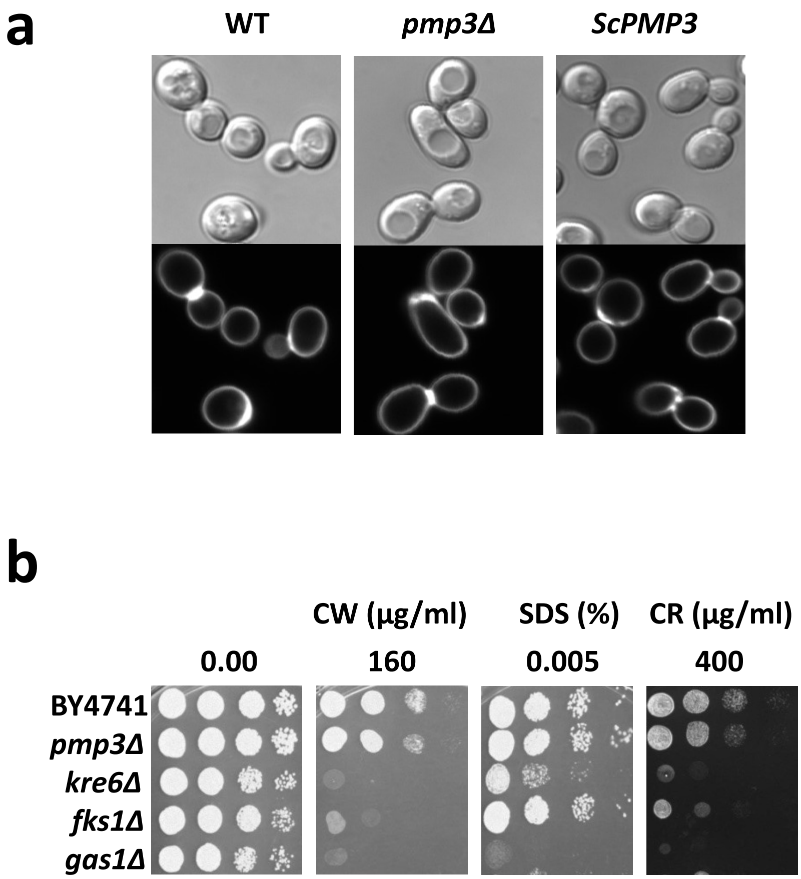

Figure S4. ***PMP3*** **deletant is not impaired in cell wall integrity.** (**a**) Chitin deposition in *PMP3* deletant is comparable to wild type (WT) and *ScPMP3* overexpression strains. Chitin was stained by calcofluor white and visualized by fluorescence microscopy (bottom panels). Top panels show DIC images of cells. (**b**) *PMP3* deletant is not sensitive to cell wall disrupting agents calcofluor white (CW), sodium dodecyl sulfate (SDS) and congo red (CR), compared to parent strain BY4741. Deletants of *KRE6*, *FKS1* and *GAS1* genes, which are known to be impaired in cell wall integrity, serve as positive controls.

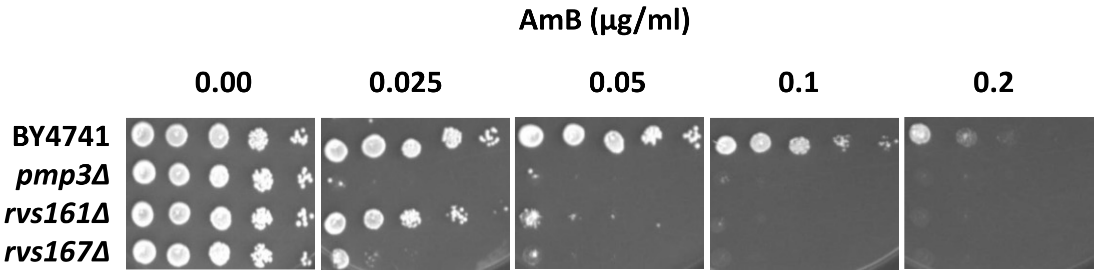

Figure S5. Sensitivity of *rvs161Δ and rvs167Δ* strains to amphotericin B. Parent (BY4741) and *pmp3Δ* strains serve as controls.

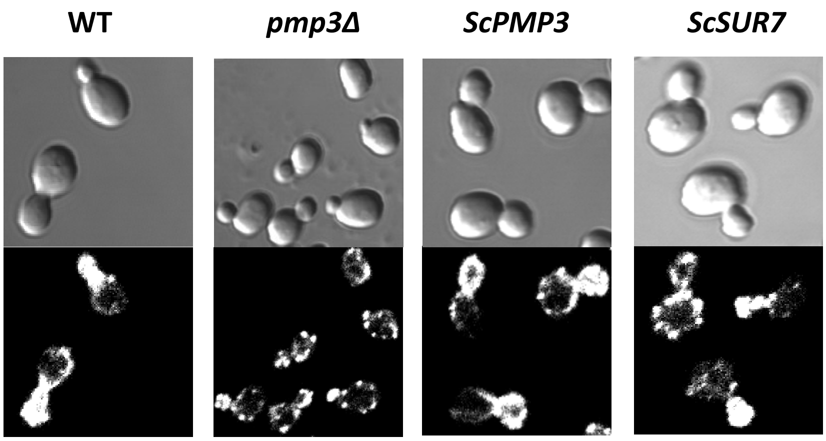

Figure S6. Representative images of actin morphology in wild-type (WT), *pmp3Δ,* and in *pmp3Δ* strains overexpressing *ScPMP3* or *ScSUR7* (bottom panels). Top panels are DIC images of corresponding fields of cells.

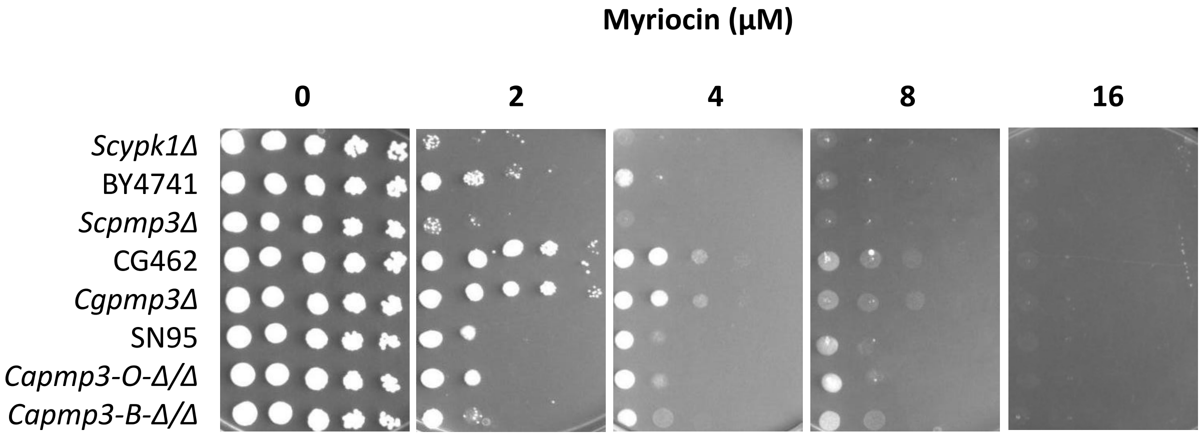

Figure S7. Effect of myriocin on *S. cerevisiae, C. glabrata and C. albicans* strains deleted in *PMP3* with respect to their respective parent strains BY4741, CG462 and SN95. For *C. albicans*, deletants of both *PMP3* ortholog (*Capmp3-O-Δ/Δ*) and best hit (*Capmp3-B-Δ/Δ)* are shown. A *S. cerevisiae* strain deleted in *YPK1* (*Scypk1Δ*), which is known to be myriocin sensitive, serves as a positive control.

Table S1. List of *S. cerevisiae* and *Candida* strains

**Strain**	**Description/Genotype**	**Reference / Source**
S. *cerevisiae* FY3	*MAT**a** ura3-52*	[1]
S. *cerevisiae* BY4741	*MAT**a** his3Δ1 leu2Δ0 met15Δ0 ura3Δ0*	[1]
S. *cerevisiae pmp3Δ*	*BY4741; YDR276C::KanMX4*	[2]
S. cerevisiae erg6Δ	*BY4741; YML008C::KanMX4*	[2]
S. *cerevisiae* BWY3818	SEY6210; Mup1-pHluorin::KAN	[3]
S. *cerevisiae* BWY3818 *pmp3Δ*	SEY6210; Mup1-pHluorin::KAN, *pmp3Δ::HIS3*	This study
C. albicans SC5314	Wild-type clinical isolate	[4]
C. albicans SN95	*arg4Δ/arg4Δ his1Δ/his1Δ URA3/ura3::imm434 IRO1/iro1::imm434*	[5]
C. albicans Capmp3-OΔ/Δ	As SN95, *orf19.1655.3Δ/orf19.1655.3Δ*	This study
C. albicans Capmp3-BΔ/Δ	As SN95, *orf19.2959.1Δ/orf19.2959.1Δ*	This study
C. glabrata CG462	BG2, Wild-type clinical isolate	[6]
C. glabrata Cgpmp3Δ	As BG2, *CAGL0M08552gΔ*	This study

Table S2. List of plasmids

Plasmid	Details	Source
pFL	pFL44L, yeast episomal plasmid with *URA3* marker	[7]
pFL-PMP3	pFL44L-*ScPMP3*	This study
pFL-OCS	pFL44L-*CaPMP3*-ortholog	This study
pFL-BCS	pFL44L-*CaPMP3*-best hit	This study
pFL-GCS	pFL44L-*CgPMP3*	This study
pFL-SUR7	pFL44L-*ScSUR7*	This study
pHAH2	*HAH2* cassette	[8]
pRS423	pRS423, yeast episomal plasmid with *HIS3* marker	[9]
pMAL2-Cre	*MAL2-Cre-SAT1* cassette	[8]
pCR2.1-NAT	*CaNAT1*[10] cassette in pCR2.1 vector	[11]

Table S3. List of oligonucleotides

**Oligos name**	**Sequence (5' to 3')**	**Use**
CaPMP3-OCS1	CTAAACAGCACAGCACAATACAACAATGAATTCTGAAAAGATTATTG	Cloning of *CaPMP3*-Ortholog ORF in place of *ScPMP3* ORF in pFL-*PMP3*
CaPMP3-OCA1	GATAGTTACTTTCGTTTATTGAATGAGCTTAATCTTTCAAAACAACG	
CaPMP3-BCS1	CTAAACAGCACAGCACAATACAACAATGCCATTCACGTGTTCC	Cloning of *CaPMP3*-Best hit ORF in place of *ScPMP3* ORF in pFL-*PMP3*
CaPMP3-BCA1	GATAGTTACTTTCGTTTATTGAATGAGCCTAGTACTTTAGAATGAC	
CgPMP3-OCS1	CTAAACAGCACAGCACAATACAACAATGGATTCAACCAAGATCGTC	Cloning of *CgPMP3* ORF in place of *ScPMP3* ORF in pFL-*PMP3*
CgPMP3-OCA1	GATAGTTACTTTCGTTTATTGAATGAGCTTAGTCGGTCAACACAAC	
CaPMP3-O-US1	CAGTTATGAGACTCACGGTACG	PCR amplification of 469 bp upstream flanking homology region for deletion of *CaPMP3*-ortholog
CaPMP3-O-UA1	GACCTGCAGCGTACGAAGAATTAATGGTGTATGTGGGATTG	
CaPMP3-O-DS1	CTCGAATTCATCGATGATATCAGACAAACACCAGAGATTTACTGC	PCR amplification of 493 bp downstream flanking homology region for deletion of *CaPMP3*-ortholog
CaPMP3-O-DA1	CGAGGCACGTGATAGACATAG	
CaPMP3-B-US1	GTAAGGGGAAGAGAGGCG	PCR amplification of 461 bp upstream flanking homology region for deletion of *CaPMP3*-best hit
CaPMP3-B-UA1	GACCTGCAGCGTACGAAGTTTGATAGTAAAATGTACTTGTAAG	
CaPMP3-B-DS1	CTCGAATTCATCGATGATATCAGCGGTCATATAACAAGGTAAATG	PCR amplification of 513 bp downstream flanking homology region for deletion of *CaPMP3*-best hit
CaPMP3-B-DA1	AAGTTCTCAGTCGCGTGTAG	
CaARG4-F61	GGTGCCACTGATCCATTG	For fusion of downstream

		flanking homology region of *CaPMP3*-ortholog or *CaPMP3*-best hit with *HAH2* cassette for gene deletion
CaARG4-R1130	GCCAACATATCCATAGTTAAAGC	For fusion of upstream flanking homology region of *CaPMP3*-ortholog or *CaPMP3*-best hit with *HAH2* cassette for gene deletion
CaPMP3-O-US2	GAACTAATCATTGGTAATGCTGG	Upstream diagnostic PCR for targeted integration of *HAH2* cassette at *CaPMP3*-ortholog
CaPMP3-O-DA2	GTTAAGGTTTTCGGTGACG	Downstream diagnostic PCR for targeted integration of *HAH2* cassette at *CaPMP3*-ortholog
CaPMP3-BDG-US2	TAGTTTGGCAAAGGTTATGG	Upstream diagnostic PCR for targeted integration of *HAH2* cassette at *CaPMP3*-best hit
CaPMP3-BDG-DA2	AAATAATGAGCTAAAGGC	Downstream diagnostic PCR for targeted integration of *HAH2* cassette in *CaPMP3*-best hit
CaPMP3-O-S3	AAATTCCGAACAGTCCTTTCG	Diagnostic PCR for deletion of *CaPMP3*-ortholog
CaPMP3-O-A3	GGGAAGACAGTGGTAGTAATGG	
CaPMP3-B-S3	TCAATTCCCCCTAAGTAGTCTC	Diagnostic PCR for deletion of *CaPMP3*- best hit
CaPMP3-B-A3	CAATCTCTACAACACAATACCAGC	
ScSUR7-OCS1	CGCAATTCGAGCTCGGTAGTCGCAGTCCTATTGTATTCTTC	PCR amplification of *ScSUR7* gene (ORF along with its promoter and terminator; +568 to -326 bp) for cloning in pFL44L
ScSUR7-OCA1	GCAGGTCGACTCTAGAGGATCCCATTAGGCACGGTTGAGAC	
M13-F	GTTTTCCCAGTCACGACG	Sequencing
M13-R	AGCGGATAACAATTTCACACAGGA	
ScPMP3-HIS3-	ATTATACATTTTGAACTAAACAGCACAGCACA	Disruption of *ScPMP3* with *HIS3*

S1	ATACAACAAACACAGTCCTTTCCCGC	marker
ScPMP3-HIS3-A1	CTTATTTCATTTGATAGTTACTTTCGTTTATTGAATGAGCGCCTCGTTCAGAATGACAC	
ScHIS3-DG-S1	TAGGAGATCTCTCTTGCGAG	Diagnostic PCR for deletion of *ScPMP3* with *HIS3* marker
ScHIS3-DG-A1	TGCACTCAACGATTAGCG	
ScPMP3-PRODG-S1	CTCAAAAGGGGGTCCTGG	
ScPMP3-TERDG-A1	CACACGTAGTTAAAATTAACAGCG	
CgPMP3-US1	GATTTATTGGTTAGCCCCTTAC	PCR amplification of 508bp upstream flanking homology region for deletion of CgPMP3
CgPMP3-UA1	CTAGCAGCGGAAGGGCTTCTGTTTTATAATGTGTGAGTG	
CgPMP3-DS1	AACTTCGTCAGCGGCCGGAACTTTCAATAACCCAACA	PCR amplification of 472bp downstream flanking homology region for deletion of CgPMP3
CgPMP3-DA1	TTTCTCTCAATTGTGGGTATATG	
CgPMP3-DG-US2	CTTTTGTTTCGAACATTCC	Diagnostic PCR for deletion of CgPMP3
CgPMP3-DG-DA2	ACTGATCTCAATAAAACCT	
CaNAT1-DS-F1	GCTTTGGATGGTTCTTTCAC	For fusion of upstream flanking homology region of CgPMP3 with *CaNAT1* cassette for gene deletion
CaNAT1-US-R1	CTATAAGCATGAATAGCTGGAGC	For fusion of downstream flanking homology region of CgPMP3 with *CaNAT1* cassette for gene deletion

Notes:

Underlined sequences in CaPMP3-OCS1, CaPMP3-BCS1 and CgPMP3-OCS1 correspond to -1 to -25 bases upstream of *ScPMP3* ORF.

Underlined sequences in CaPMP3-OCA1, CaPMP3-BCA1 and CgPMP3-OCA1 correspond to +1 to +28 bases downstream of *ScPMP3* ORF.

Underlined sequences in CaPMP3-O-UA1 and CaPMP3-B-UA1 correspond to bases 1 to 18 of *HAH2* cassette[8].

Underlined sequences in CaPMP3-O-DS1 and CaPMP3-B-DS1 correspond to bases 3784 to 3806 of *HAH2* cassette.

Underlined sequences in ScSUR7-OCS1 and ScSUR7-OCA1 correspond to bases 406 to 423 and 429 to 450, respectively, of pFL44L.

Underlined sequences in ScPMP3-HIS3-S1 and ScPMP3-HIS3-A1 correspond to 40 bases upstream and downstream, respectively, of *ScPMP3* ORF.

Underlined sequences in CgPMP3-UA1 and CgPMP3-DS1 correspond to bases 1 to 16 and 1305 to 1320, respectively, flanking *CaNAT1* cassette in pCR2.1 vector[11].

REFERENCES

1 Winston, F., Dollard, C. & Ricupero-Hovasse, S. L. Construction of a set of convenient *Saccharomyces cerevisiae* strains that are isogenic to S288C. *Yeast* **11**, 53-55 (1995).

2 Giaever, G. *et al.* Functional profiling of the *Saccharomyces cerevisiae* genome. *Nature* **418**, 387-391 (2002).

3 Prosser, D. C., Whitworth, K. & Wendland, B. Quantitative analysis of endocytosis with cytoplasmic pHluorin chimeras. *Traffic* **11**, 1141-1150 (2010).

4 Fonzi, W. A. & Irwin, M. Y. Isogenic strain construction and gene mapping in *Candida albicans*. *Genetics* **134**, 717-728 (1993).

5 Miller, J. P. *et al.* Large-scale identification of yeast integral membrane protein interactions. *Proc Natl Acad Sci U S A* **102**, 12123-12128 (2005).

6 Cormack, B. P., Ghori, N. & Falkow, S. An adhesin of the yeast pathogen *Candida glabrata* mediating adherence to human epithelial cells. *Science* **285**, 578-582 (1999).

7 Stettler, S. *et al.* A general suppressor of RNA polymerase I, II and III mutations in *Saccharomyces cerevisiae*. *Mol Gen Genet* **239**, 169-176 (1993).

8 Sharma, S. *et al.* Sphingolipid biosynthetic pathway genes *FEN1* and *SUR4* modulate amphotericin B resistance. *Antimicrob Agents Chemother* **58**, 2409-2414 (2014).

9 Christianson, T. W., Sikorski, R. S., Dante, M., Shero, J. H. & Hieter, P. Multifunctional yeast high-copy-number shuttle vectors. *Gene* **110**, 119-122 (1992).

10 Shen, J., Guo, W. & Kohler, J. R. *CaNAT1*, a heterologous dominant selectable marker for transformation of *Candida albicans* and other pathogenic *Candida* species. *Infect Immun* **73**, 1239-1242 (2005).

11 Green, B., Bouchier, C., Fairhead, C., Craig, N. L. & Cormack, B. P. Insertion site preference of Mu, Tn5, and Tn7 transposons. *Mob DNA* **3**, 3 (2012).
